# The Role of Bioenergetics in Neurodegeneration

**DOI:** 10.3390/ijms23169212

**Published:** 2022-08-16

**Authors:** Taylor A. Strope, Cole J. Birky, Heather M. Wilkins

**Affiliations:** 1 University of Kansas Alzheimer’s Disease Center, Kansas City, KS 66205, USA; 2 Department of Biochemistry and Molecular Biology, University of Kansas Medical Center, 3901 Rainbow Blvd, Kansas City, KS 66160, USA; 3 Department of Neurology, University of Kansas Medical Center, 3901 Rainbow Blvd, Kansas City, KS 66160, USA

**Keywords:** mitochondria, bioenergetics, Alzheimer’s disease, Parkinson’s disease, amyotrophic lateral sclerosis

## Abstract

Bioenergetic and mitochondrial dysfunction are common hallmarks of neurodegenerative diseases. Decades of research describe how genetic and environmental factors initiate changes in mitochondria and bioenergetics across Alzheimer’s disease (AD), Parkinson’s disease (PD), and amyotrophic lateral sclerosis (ALS). Mitochondria control many cellular processes, including proteostasis, inflammation, and cell survival/death. These cellular processes and pathologies are common across neurodegenerative diseases. Evidence suggests that mitochondria and bioenergetic disruption may drive pathological changes, placing mitochondria as an upstream causative factor in neurodegenerative disease onset and progression. Here, we discuss evidence of mitochondrial and bioenergetic dysfunction in neurodegenerative diseases and address how mitochondria can drive common pathological features of these diseases.

## 1. Introduction

Neurodegenerative diseases have common pathological mechanisms, including inflammation, neuronal loss, vascular changes, blood brain barrier disruption, protein aggregation/loss of proteostasis, and bioenergetic dysfunction [1,2]. While there are many pathologies in neurodegenerative diseases, bioenergetic dysfunction is often cited as a causative factor [3,4]. Bioenergetic dysfunction is an increasingly recognized driver of the progression and onset of neurodegenerative diseases. This is not surprising, given the unique bioenergetic considerations of the brain and central nervous system (CNS). The brain utilizes approximately 20% of the body’s energy output while only comprising approximately 2% of total body weight [5]. The brain is an energy-expensive organ and is very susceptible to disruptions in bioenergetic function [2]. 

Neurons and astrocytes have unique bioenergetic coupling relationships that are disturbed in neurodegenerative diseases [6,7]. The failure of bioenergetic pathways is attributed to mitochondrial dysfunction, which can be a result of inherited mitochondrial DNA (mtDNA) heteroplasmy, acquired mtDNA mutations during aging, and/or environmental factors [4]. Mitochondrial dysfunction is hypothesized to drive other pathological mechanisms, including inflammation, vascular abnormalities, proteostasis, and neuronal loss. 

Mitochondria play a major role in metabolic processes in the brain, where the continuous production of ATP (adenosine triphosphate) is required [8]. In the citric-acid cycle (TCA), acetyl CoA is oxidized to generate electron carriers for use in the electron transport chain (ETC). These electron carriers, NADH and FADH_2_, are oxidized, and energy is harnessed to pump protons out of the mitochondrial matrix as their electrons pass through enzymatic membrane complexes [9]. The proton gradient generated in the ETC allows ATP synthase to catalyze the synthesis of ATP from ADP and phosphate. This highly efficient process is known as oxidative phosphorylation. In addition to their metabolic functions, mitochondria serve as vital regulators of apoptosis, calcium levels, inflammation, proteostasis, and the biosynthesis of macromolecules. Mitochondria are important for cellular function, but dysfunction in mitochondria has also proved to be important in the pathogenesis of neurodegenerative diseases. Here, we discuss and review the literature covering bioenergetic and mitochondrial dysfunction in Alzheimer’s disease (AD), Parkinson’s disease (PD), and amyotrophic lateral sclerosis/frontotemporal lobar dementia (ALS/FTD). We further discuss how bioenergetic/mitochondrial dysfunction can lead to common pathological findings in these diseases. 

## 2. Bioenergetics and Neurodegeneration

### 2.1. Alzheimer’s Disease

AD is the most common form of dementia and is characterized by the accumulation of intraneuronal neurofibrillary tangles and amyloid plaques in the brain. Tangles are composed of hyperphosphorylated tau, while amyloid plaques are insoluble extracellular aggregates of amyloid beta (Aβ) [10]. Aβ is generated from the cleavage of amyloid-precursor protein (APP) by secretase enzymes. Historically, the amyloid-cascade hypothesis has dominated the field of AD research and therapeutic avenues. Given the most recent clinical trial failures, more interest is being placed in alternative hypotheses for AD, including bioenergetic and mitochondrial dysfunction [11]. 

AD cases are categorized as either familial or sporadic. Familial forms of AD account for approximately 5% of all cases and are caused by mutations or duplications in *APP* (chromosome 21), or mutations in the secretase enzymes presenilin 1 (*PS1*, on chromosome 14) or presenilin 2 (*PS2* on chromosome 1). Sporadic AD accounts for most cases (~95%) and risk factors include age, diabetes, cardiovascular disease, education level, maternal history, and polymorphisms in the Apolipoprotein E (*APOE,* chromosome 19) gene [12]. Recent genome-wide association studies (GWAS) implicate risk associated with single-nucleotide polymorphisms (SNP) in genes that encode for pathways affecting pathologies including inflammation and mitochondrial/bioenergetic function [13,14].

**Figure 1:**
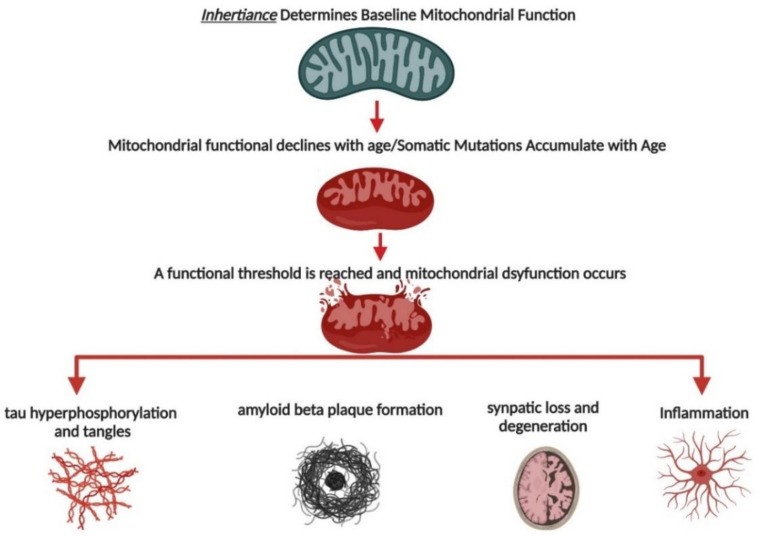
Mitochondrial-Cascade Hypothesis of AD. Inherited mtDNA determines baseline function for individuals. During aging, mitochondrial function declines and somatic mutations accumulate. If baseline function is low, mitochondrial dysfunction occurs earlier than if baseline function is high. Eventually, a functional threshold is reached where mitochondrial dysfunction leads to a cascade of loss of proteostasis (Aβ plaques, tau tangles), inflammation, and neuronal loss/degeneration.

AD is a complex, multifaceted disease with numerous molecular pathologies. In a triple-transgenic AD female mouse model (3xTg-AD), mitochondrial deficits preceded AD pathology [15]. This study (and others reviewed below) support the mitochondrial-cascade hypothesis of AD, which states that mitochondrial dysfunction is the cause of AD onset and progression [3,4]. The mitochondrial-cascade hypothesis, developed by Dr. Russell Swerdlow, postulates that inherited mtDNA determines baseline mitochondrial function and that, throughout aging, mitochondrial function declines. Some individuals inherit mtDNA, which allows high-baseline mitochondrial function and protects against AD onset and progression. However, in some individuals, inherited mtDNA causes lower baseline mitochondrial dysfunction and, during aging, a threshold is reached that leads to AD onset and progression. Essentially, this hypothesis places mitochondria as the first mechanism in the cascade of AD pathologies, including those listed in the introduction (Figure 1). 

**Figure 2:**
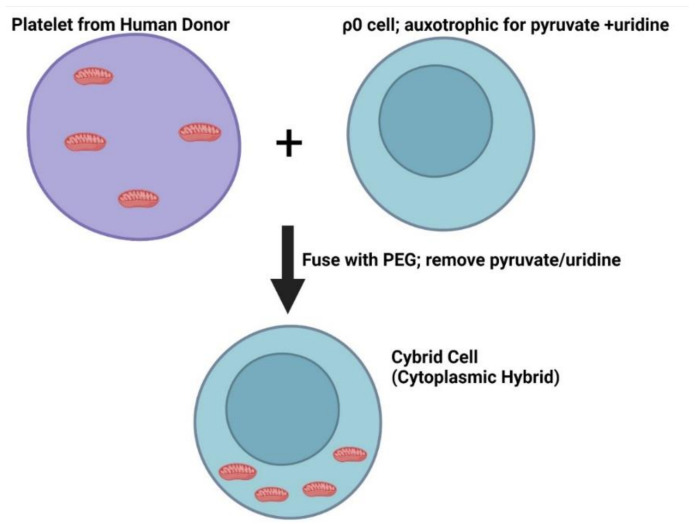
Cytoplasmic Hybrids (Cybrids). Patient-derived platelets with mitochondria are fused with cells that lack mtDNA (ρ0 cells) using PEG (polyethylene glycol). The ρ0 cells are auxotrophic for pyruvate and uridine. After fusing platelets with ρ0 cells, pyruvate and uridine are withdrawn to select for cells that received mitochondria.

Cytoplasmic hybrid, or cybrid studies, further place mtDNA inheritance and mitochondrial dysfunction upstream of other AD pathologies. Cybrids are hybrid cells in which patient-derived platelets are fused with cells that do not contain mtDNA. This generates cells with mtDNA from patients on a consistent nuclear DNA background (Figure 2). The purpose of cybrids is to study the influence of mtDNA on cell function and disease pathologies [16]. In a cybrid AD model, altered bioenergetic function and bioenergetics-associated infrastructures were observed [17]. This included changes in oxygen consumption, respiratory coupling, and glucose utilization. 

AD has been heavily linked with oxidative stress, a consequence of bioenergetic processes [18]. In low concentrations, reactive oxygen species (ROS) are used as cell-signaling molecules. ROS become harmful in situations in which antioxidants and redox systems are depleted, such as in aging and with mitochondrial dysfunction. Oxidative stress in the brain is increased with both aging and AD [19]. In postmortem AD brain tissue, oxidative damage to mtDNA is increased, which can lead to mitochondrial dysfunction [20]. The disruption of oxidative phosphorylation and ETC complexes is observed in AD and can increase ROS production. ATP synthase activity is reduced in AD postmortem brains when compared to control cases, and this enzyme can be disrupted through ROS-associated damage [15].

Beyond ROS and changes to mtDNA, AD subjects have reduced cytochrome oxidase (COX, or complex IV) Vmax and flux [21,22]. This deficit has been observed in brain tissue, blood, muscle, and skin [23,24]. These data support the role of mitochondrial dysfunction in driving AD pathology and not vice versa, because systemic tissues lack amyloid plaques and neurofibrillary tangles, but mitochondrial deficits are present. Further studies directly support mitochondrial dysfunction in modulating tau phosphorylation, Aβ production, and the aggregation of both [25,26,27]. 

Novel neuroimaging studies have allowed enhanced studies examining the role of AD pathologies and energy metabolism in the cognition of human subjects. For example, studies correlate decreased cerebral oxygen with the severity of dementia [28,29]. Specialized magnetic resonance imaging (MRI) studies have shown that phosphate metabolism and metabolites are reduced in subjects with amnestic mild cognitive impairment (aMCI), a diagnosis that precedes AD [30]. Brain-glucose metabolism is reduced in AD subjects, a finding that was first described in the 1980s [31]. Current research suggests reduced brain-glucose metabolism, measured by fluorodeoxyglucose positron emission tomography (FDG-PET), occurs early in the disease process, and can be detected in those at highest risk for AD [32]. 

Decades of research record mitochondrial and bioenergetic dysfunction in AD. Studies support the role of altered energy metabolism preceding other common AD pathological hallmarks. These studies are vitro, in vivo, and on human subjects, and they are consistent. Bioenergetic and mitochondrial dysfunction should be high-priority research areas in AD for future therapeutic efforts. 

### 2.2. Parkinson’s Disease

PD is characterized by the degeneration of dopaminergic neurons in the substantia nigra of the basal ganglia. Dopaminergic neurons in the substantia nigra form the nigrostriatal pathway, a major dopamine pathway in the brain responsible for voluntary movement. PD is often recognized by the development of Lewy bodies in the brain, mostly composed of aggregated α-synuclein protein. The main symptoms of PD include tremors, muscular stiffness, bradykinesia, and a shuffling gait. Mitochondrial dysfunction is an important factor in the development of both sporadic and familial PD. 

PD cases are categorized as either familial or sporadic. Familial forms of PD account for approximately 10–15% of all cases and are caused by familial mutations in numerous identified genes. These genes include those with autosomal-dominant patterns of inheritance, such as alpha-synuclein (*SNCA*, chromosome 4), vacuolar protein sorting 35 ortholog (*VPS35*, chromosome 8), and leucine-rich repeated kinase 2 (*LRRK2*, chromosome 12), or those with autosomal recessive inheritance, such as glucocerebrosidase (*GBA*, chromosome 1), parkin (*PARK2*, chromosome 6), DJ-1 (*PARK7*, chromosome 1), and PTEN induced kinase 1 (*PINK1*, chromosome 1). Currently, other genes are being identified in familial forms of PD; therefore, this list may not be all-encompassing [33]. Sporadic PD accounts for approximately 85–90% of cases, with risk factors including age, head injuries, and sex (its male predominate). Some environmental risk factors also include exposure to pesticides and resulting water contamination [34]. Sporadic PD has been associated with SNPs in genes which are also implicated in familial PD, as well as other rare, low-abundance gene variants from GWAS studies [35]. Overall, the genes implicated in familial and sporadic forms of PD, as well as environmental exposures, are linked to disrupted bioenergetics and mitochondrial dysfunction. 

Cells-harboring mtDNA from PD subjects, on a consistent nuclear DNA background (cybrids; Figure 2), show a direct role for mitochondrial genetics in PD pathology. PD-derived cybrids show reduced complex I (CI) flux, increased ROS and ETC proton leak, and increased mitochondrial calcium [36,37]. PD-derived cybrids also have increased oligomerization of α-synuclein and develop Lewy bodies [38]. Overall, these studies support the role of mtDNA in the disease pathologies observed in PD. 

It has been shown that mitochondrial dysfunction influences the pathology of PD, especially when considering mitochondrial CI [39,40]. CI is the initial electron acceptor of NADH in the ETC, and the disruption of this complex results in a dramatic loss of energetic capacity. A recent study has shown, in a mouse knockout model, that the disruption of CI induces PD [41]. The disruption of CI can result from oxidative damage, among other factors. The physiological importance of CI in PD has been considered for decades, following the observation that 1-methyl-4-phenyl-1,2,3,6-tetrahydropyridine (MPTP) selectively inhibits CI. First seen as a side effect of illicit drug use, MPTP has since been used to model PD in a variety of animal studies [42]. While MPTP does not cross the blood–brain barrier, its metabolite, MPP^+^, does, where it accumulates in dopaminergic neurons and induces toxicity through mitochondrial dysfunction [43]. 

**Figure 3:**
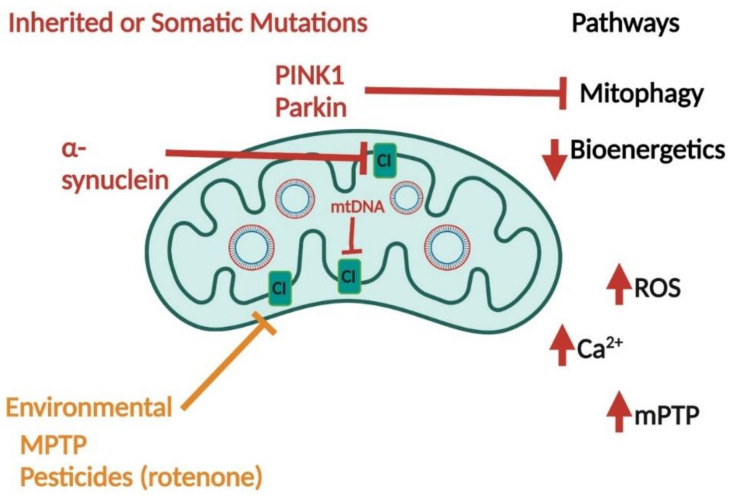
Mitochondrial Dysfunction in PD. Inherited mutations in genes encoding proteins for PINK1, Parkin, and α-synuclein affect mitochondrial function through the inhibition (red blunt arrow) of CI and/or mitophagy inhibition. Environmental factors, such as MPTP and pesticide rotenone, inhibit CI (orange blunt arrow). mtDNA (either inherited or somatic mutations) inhibit complex I (red blunt arroa). All factors lead to increased ROS, mitochondrial calcium, and the activation of the mitochondrial permeability transition pore (mPTP) with decreased bioenergetics and mitophagy.

ROS is an important factor in bioenergetic and mitochondrial dysfunction in PD. Mitochondrial monoamine oxidases function in dopamine turnover, a major factor in PD-symptom onset [44]. The reaction of monoamine oxidases generates H_2_O_2_, which, in large amounts, can lead to cell toxicity [45]. Monoamine oxidases are involved in the clearance of 6-hydroxydomaine (6-OHDA), a commonly used dopaminergic neuron toxin for PD research [46]. In addition, 6-OHDA interacts with the ETC, where it impairs ATP synthase and reduces mitophagy [47]. Overall, PD models use toxins that directly interact with mitochondria to induce PD phenotypes. This highlights the direct involvement of mitochondrial dysfunction in PD pathology (Figure 3). 

The genes implicated in familial PD also interact with mitochondria and induce mitochondrial dysfunction. For instance, α-Synuclein localizes to mitochondria and alters mitochondrial membrane structure and function. Furthermore, α-Synuclein interacts with outer mitochondrial membrane components, as well as ATP synthase, leading to a reduction in ATP. Toxic α-Synuclein oligomers lead to mitochondrial-permeability transition pore (mPTP) activation through an increase in ROS production and the inhibition of CI [48].

PINK1 and Parkin are mitophagy mediators. Mitophagy is the process of the phagosome removal of damaged mitochondria. Mutations in PINK1 and Parkin cause familial PD, and SNPs are also associated with sporadic forms of PD. Mitophagy impairment is observed in both forms of PD. PINK1 and Parkin interact at the outer mitochondrial membrane to facilitate autophagosome recruitment to mitochondria. Typically, PINK1 localizes to depolarized mitochondria and recruits Parkin and ubiquitin [49]. PINK1 activates Parkin and ubiquitin through phosphorylation. After Parkin is activated and accumulates at the outer mitochondrial membrane, it polyubiquitinates mitochondrial proteins, which leads to proteosome degradation [49]. Mutations in PINK1 and Parkin impair mitophagy processes and may also be associated with reductions in mitochondrial transport in neurons [50].

Genes and environmental factors that cause familial or sporadic forms of PD directly modulate mitochondrial function. In familial PD, genes that regulate mitophagy and vesicle transport are causative in the onset and progression of the disease. Further, proteins that aggregate in PD localize to mitochondria and induce dysfunction. PD is modeled using toxins that target mitochondrial CI. Overall, PD and its associated pathologies are strongly associated with mitochondrial and bioenergetic dysfunction. 

### 2.3. Amyotrophic Lateral Sclerosis and Frontotemporal Lobar Dementia 

ALS leads to the rapid degeneration of motor neurons, causing muscle spasticity, atrophy, and paralysis. Patients diagnosed with ALS survive approximately 2–5 years. Most ALS cases are sporadic (~90%), but familial ALS accounts for approximately 10% of cases. Genetic mutations in superoxide dismutase 1 (*SOD1*, chromosome 21), *C9orf72* (chromosome 9), heterogeneous nuclear ribonucleoprotein P2 (*FUS*, chromosome 16), NIMA related kinase 1 (*NEK1*, chromosome 4), Ubiquilin-2 (*UBQLN2*, chromosome X), kinesin family member 5A (*KIF5A*, chromosome 12), and TAR-DNA binding protein 43 (*TDP43*, chromosome 1) lead to familial ALS onset [51,52,53]. Both SOD1 and TDP43 localize to mitochondria and induce mitochondrial dysfunction and bioenergetic disruption. Sporadic ALS risk factors include sex (predominately male) and a prior history of head injuries. GWAS implicate pathways including autophagy, cholesterol, and vesicle trafficking as disease modifiers [54,55]. Furthermore, GWAS have shown shared pathways between AD, PD, and ALS, which includes many genes involved in metabolic pathways. 

Mitochondrial genetics may also contribute to ALS pathology. This is supported by studies of ALS-subject-derived cybrid cells (Figure 2). These models show reduced CI activity, altered ROS dynamics, and increased mitochondrial calcium [56]. To date, the effects of mtDNA inheritance on SOD1, FUS, C9orf72-derived peptides, and TDP43 aggregation or accumulation have not been studied in ALS-derived cybrid models. 

Both sporadic and familial ALS are hallmarked by loss of bioenergetic function and mitochondrial dysfunction in motor neurons. Motor neurons derived from the induced pluripotent stem cells of ALS patients show increased ROS, decreased ATP, and depolarized mitochondria [57]. Furthermore, mitochondrial dysfunction precedes ALS onset in mouse models. In the G93A *SOD1* ALS mouse model, mitochondrial dysfunction was observed prior to the onset of hanging-muscle-strength atrophy [58]. Overall, mitochondrial dysfunction could be upstream of other pathologies in ALS, driving its onset and progression. 

Mutations in the genes that cause familial ALS are associated with mitochondrial dysfunction and increased interactions/localization of mutant proteins to mitochondria. SOD1 is typically found in the cytoplasm, but mutations associated with ALS lead to mitochondrial localization [59,60,61]. In animal models of ALS, which express transgenic mutant *SOD1* constructs, mitochondrial swelling and increased calcium, membrane depolarization, decreased ATP, and reduced ETC activities are observed [62,63,64,65]. Mutant SOD1 was shown to bind to the anti-apoptotic protein, BCL-2, in mouse models and human-spinal-cord samples [61]. Cell models that express mutant *SOD1* also show mitochondrial dysfunction. These findings include mitochondrial swelling, reduced ETC function, changes to redox balance, and decreased ATP [65]. Mitochondrial dysfunction was not observed in models expressing wild-type (WT) *SOD1*. Mutations in *SOD1* elicit mitochondrial dysfunction across in vitro and in vivo models. 

TDP43 mutations are associated with both familial and sporadic forms of ALS. TDP43 regulates gene transcription in the nucleus but can form aggregates within the cytoplasm [66,67]. Both cell and animal models suggest the role of TDP43 in mitochondrial dysfunction. The overexpression of mutant and WT forms induces changes in mitochondrial mass and structure, as well as reduced mitochondrial transport in motor neurons [68]. 

Fused-in sarcoma (FUS) is an RNA-binding protein implicated in ALS/FTD pathology. FUS shuttles between the cytoplasm and the nucleus, where it regulates gene expression, RNA splicing, and mRNA processing. FUS accumulates in the cytoplasm and induces mitochondrial dysfunction [69]. FUS binds to ATP synthase within mitochondria and induces the unfolded protein response [70,71]. Other studies support the role of FUS in the dysregulation of mitochondrial and endoplasmic reticulum (ER) interactions. Overall, the genes implicated in both sporadic and familial ALS may drive disease onset and progression through mitochondrial/bioenergetic dysfunction.

Mitochondrial and bioenergetic dysfunction are observed in human ALS subjects. One study determined that DNA damage in postmortem tissue correlated with oxidative damage [72]. The mtDNA levels are reduced and there is an increase in mtDNA mutations and deletions in postmortem ALS spinal cord tissue [73]. Mitochondria from dorsal-root ganglion cells have increased protein aggregates within the intermembrane space and cristae [74]. The anterior horn of the spinal cord showed mitochondrial aggregation and altered morphology, while COX activity was reduced [75]. Mitochondrial and bioenergetic changes are also observed in muscle from ALS patients. Findings in skeletal muscle include reduced activities for CI, COX, citrate synthase (CS), and succinate dehydrogenase [76]. Mitochondrial morphology and mtDNA deletions and mutations are also observed in muscle [77]. Mitochondrial changes are evident across in vitro and in vivo models and human ALS subjects within spinal cord and muscle. 

ALS is often diagnosed with FTD, and it is currently estimated that 50% of those afflicted with ALS also develop FTD [78]. FTD is associated with neuronal loss in the frontal and temporal lobes, leading to disinhibition, changes in personality, and speech pathology. Mitochondrial dysfunction is a hallmark of ALS/FTD and the *C9orf72* gene is associated with ALS/FTD. Increased GGGGCC repeat expansions in *C9orf72* lead to the production of dipeptide repeat proteins (DPR). DPR (GR)_80_ preferentially binds mitochondria ribosomal proteins, causing mitochondrial dysfunction in induced pluripotent stem-cell-derived neurons [79]. Other genetic risk factors are conserved between ALS and FTD and implicate mitochondrial function, as discussed above.

**Figure 4:**
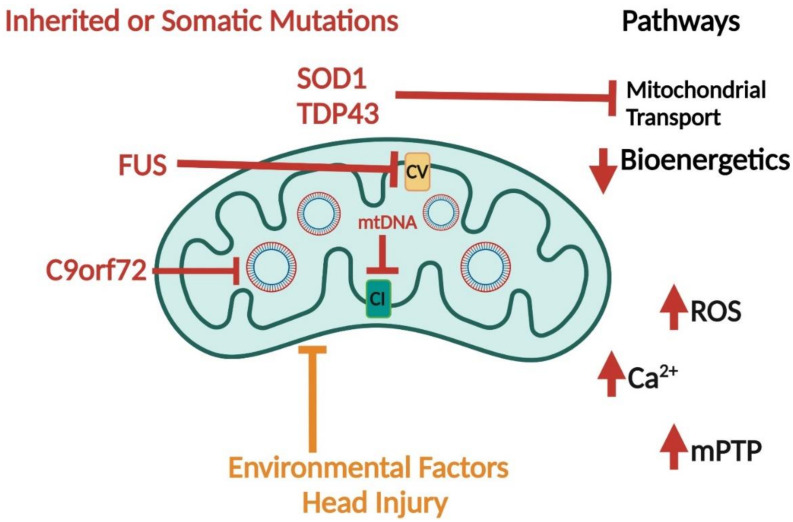
Mitochondrial dysfunction in ALS. Inherited mutations in genes encoding proteins for TDP43 and SOD1 affect mitochondrial function through inhibition of bioenergetics and mitochondrial transport. Mutations in FUS inhibit ATP synthase or complex V (CV) and C9orf72 peptides interact with mitochondrial ribosomes and affect the expression of mtDNA encoded proteins (red blunt arrows). Environmental factors and head injuries affect overall mitochondrial function (orange blunt arrow). Furthermore, mtDNA (either inherited or somatic mutations) inhibits complex I (red blunt arrow). All these factors lead to increased ROS, mitochondrial calcium, and activation of the mitochondrial permeability transition pore (mPTP) with decreased bioenergetics.

ALS and FTD are associated with significant changes in mitochondrial and bioenergetic function. The mechanisms of mitochondrial functional changes in driving the diseases’ pathology are not completely understood, but data support the upstream role of mitochondria in the disease process (Figure 4). 

## 3. Consequences of Disturbed Bioenergetics

Numerous studies place mitochondrial dysfunction and bioenergetic stress upstream of other pathological features of neurodegenerative diseases. Here, we discuss how mitochondrial and bioenergetic dysfunction can lead to the pathological hallmarks that are common between AD, PD, ALS, and ALS/FTD. 

### 3.1. Neuronal Loss and Degeneration 

The mechanism(s) of neuronal loss are not fully understood. Neuronal loss and degeneration have been difficult to study, as animal models of disease (such as AD) do not have neurodegenerative features as in those observed in human disease. Another major caveat in the understanding of neuronal loss and degeneration is a lack of specific markers and harmonized methods to measure this phenomenon [80]. Several mechanisms have been identified in neuronal loss and degeneration, and one of the most cited is oxidative stress [81].

ROS generation is a by-product of energy production, particularly at the ETC. Oxidative stress occurs when ROS production overwhelms antioxidant defense mechanisms, or those mechanisms become depleted [82]. The consequences of oxidative stress include DNA damage to chromosomes in the nucleus, as well as mtDNA damage; mtDNA is particularly prone to oxidative damage due to its lack of histones and proximity to the ETC [83,84]. 

Reduced mitophagy or loss of mitophagy function are hallmarks of neurodegenerative diseases and can lead to neuronal cell death [85]. The exact mechanism(s) of mitophagy failure are not understood, but mitophagy is currently a major therapeutic target. Studies in models of AD showed significant results when mitophagy was increased [86,87,88,89,90]. However, without a full understanding of where or how mitophagy is disrupted, it will be difficult to appropriately target. For example, the lesion affecting the mitophagy could originate during induction or lysosome function. If lysosome function is disrupted, then inducing mitophagy pathways at the level of increasing mitochondrial trafficking to lysosomes is not advantageous. 

Reduced mitochondrial trafficking can lead to bioenergetic dysfunction at synapses and neuronal cell death [91,92,93,94]. For proper synaptic signaling, a large amount of ATP is required. Mitochondria are important for the generation of neurotransmitters and providing energy for their release and uptake. One hypothesis is that the reduced mitochondrial numbers in synapses can lead to reduced signaling and the degeneration of neurons. Furthermore, the mechanisms of mitophagy and the mitochondrial biogenesis within synapses are important factors that require further study. Some mechanisms have been described recently, including transcellular mitophagy [95]. Transcellular mitophagy is a process in which neurons release mitochondria into the synapse, resulting in phagocytosis and mitophagy by glial cells. Limited research is available on this process, but it remains a mechanism that could link mitophagy disruption and synaptic dysfunction, beyond bioenergetic loss. 

Disturbed mitochondrial bioenergetics lead to altered neurometabolic coupling, energy processing, and function [96]. Neurons are non-mitotic, and some neurons are not replaced throughout the lifespan. Some studies do support neurogenesis (new neuron formation) in certain brain structures, including the hippocampus, but others dispute these findings. Regardless, neurons are not easily replaced if they degenerate. 

The bioenergetic requirements of neurons are high, especially for synaptic function and neurotransmitter synthesis, release, and uptake. Oxidative phosphorylation accounts for the majority of neuronal ATP production and, upon disruption, can have severe consequences. Neurons do not have the ability to switch to glycolytic or non-aerobic ATP production because they lack key enzymes for this pathway [97,98,99]. As a result, neurons rely on astrocytes for bioenergetic support, especially as a source for lactate. During stress, astrocytes can increase the glycolytic output of lactate to support neurons [1,98,99,100,101,102]. A consequence of these bioenergetic shifts, however, is an imbalance in lactate/glucose levels, which can alter mitochondrial function and the redox pairs that control bioenergetic-pathway feedback [103]. These effects can lead to further mitochondrial damage, glutamate toxicity, and neurodegeneration [100,103]. 

### 3.2. Proteostasis

Recent studies implicate mitochondria and mitophagy in the regulation of proteostasis. In vitro work describes mitochondria as guardians of the cytoplasm (MAGIC), where aggregation-prone proteins localize to mitochondria and are disposed of via mitophagy [104]. Beyond their direct involvement in protein folding and proteostasis pathways, mitochondria may play a direct role in modulating the expression and production of aggregation-prone proteins. 

Decades of research link mitochondrial and bioenergetic stress with increased or decreased APP processing to Aβ [27,105]. More recently, mitochondrial membrane potential and, ultimately, mitochondrial activity were shown to directly influence the secretion of Aβ and the accumulation of intracellular Aβ [27]. Studies also directly link changes to mitochondrial function driving tau phosphorylation and accumulation [25]. While these studies are more relevant to AD, further studies are warranted to examine aggregation-prone proteins in PD and ALS. Most studies focused on the effects of α-synuclein, SOD1, or TDP43 on mitochondrial function, but the effects of mitochondrial function on the localization and aggregation of these proteins have not been studied. 

Mitochondria contain quality-control mechanisms to maintain proteostasis. Energy disruption can negatively influence protein folding. During protein-unfolding stress responses, mitochondria activate the mitochondrial unfolded-protein response pathway (UPR^mt^). In many neurodegenerative disease models, these pathways are activated (CHOP, ATF4/5, AFS-1; see cited review) [106,107]. While the UPR^mt^ promotes the recovery of mitochondria, its constant activation can lead to a state of chronic mitochondrial recovery and imbalanced bioenergetics. The role that mitochondrial dysfunction plays in driving UPR^mt^ versus other neurodegenerative pathologies (such as disrupted proteostasis) is currently unclear. 

### 3.3. Inflammation 

Damage associate molecular patterns (DAMPs) are molecules released during cell death or damage [108,109,110]. DAMPs induce an inflammatory response, probably due to their resemblance of virus- or bacteria-derived particles. Mitochondria and mitochondrial components can serve as DAMPs [111,112]. Mitochondria-induced inflammation can lead to changes in AD-like pathology in vitro and in vivo [111,113]. Further work is needed to determine whether this is a driving factor in neurodegenerative diseases, or whether these findings are specific to AD pathologies.

Mitochondrial dysfunction can directly or indirectly induce inflammation. Mitochondrial dysfunction can lead to necrotic cell death, the release of cell components, and the activation of inflammation through DAMP pathways [112]. Mitochondria can also activate inflammation via redox through the NLRP3 inflammasome. The release of mtDNA fragments to the cytoplasm activates the cGAS-STING pathway, TLR9/NFκB inflammatory pathways, and cytokine production [108,114]. Overall, mitochondria-induced inflammation is observed, and these pathways are activated in neurodegenerative diseases [112,115].

## 4. Concluding Remarks

Mitochondrial and bioenergetic changes are common across AD, PD, and ALS/FTD. These changes were discovered decades previously; however, its mechanisms are not fully understood. The data are consistent across in vitro, in vivo, and human-derived samples, which show specific changes in mitochondrial function across neurodegenerative diseases. The mitochondrial changes observed in neurodegenerative diseases are linked to mtDNA inheritance or somatic mutations. In some cases, mtDNA SNPs can modulate the risk of neurodegenerative disease and interact with nuclear-encoded genetic risk factors.

Recent research has revealed a strong correlation between mitochondrial changes in disease and observed pathological hallmarks. Mitochondria are implicated in pathological changes, including loss of proteostasis, inflammation, and cell loss/death. As models for neurodegenerative diseases improve and further GWAS are completed, we can better understand the role of mitochondria in the onset and progression of neurodegenerative diseases.

There is a significant need for further research in order to determine how mitochondrial and bioenergetics lead to neurodegenerative phenotypes. Many clinical trials target mitochondria and bioenergetics (both directly and indirectly) for neurodegenerative diseases. Based on this, a better understanding of the underlying mechanisms will help in the design of better therapeutics and biomarkers for target engagement outcomes. The data are very clear that mitochondria are likely upstream in the pathological process of neurodegeneration; further research will aid in understanding how and why.

## Data Availability

Not applicable.
